# Inhibition of SENP1 induces radiosensitization in lung cancer cells

**DOI:** 10.3892/etm.2013.1259

**Published:** 2013-08-08

**Authors:** RUO-TIAN WANG, XIU-YI ZHI, YI ZHANG, JIAN ZHANG

**Affiliations:** Department of Thoracic Surgery, Xuanwu Hospital of Capital Medical University (CMU), Beijing 100053, P.R. China

**Keywords:** lung carcinoma, radiotherapy, radiosensitivity, SENP1

## Abstract

Lung cancer is one of the most common and lethal types of malignancy. To date, radiotherapy and chemotherapy have been used as the two major treatment methods. However, radioresistance of lung cancer remains a therapeutic hindrance. The aim of this study was to identify whether small ubiquitin-related modifier (SUMO)-specific protease 1 (SENP1) is a marker of radioresistance that may serve as a target for enhancing the efficacy of lung carcinoma radiotherapy. SENP1 was observed to be overexpressed in lung cancer tissues, and the modulation of SENP1 expression was demonstrated to significantly affect the proliferation of lung cancer cells. Moreover, silencing the expression of SENP1 using small interfering RNA (siRNA) significantly sensitized lung cancer cells to radiation. Mechanically, it was demonstrated that SENP1 depletion significantly enhanced ionizing radiation (IR)-induced cell cycle arrest, γ-H2AX expression and apoptosis. Thus, these data suggest that SENP1 may be a desirable drug target for lung carcinoma radiotherapy.

## Introduction

Lung carcinoma is one of the most common and lethal types of malignancy. Due to the high occurrence and mortality rates of lung cancer, the treatment of the disease has always been a significant issue (1). Furthermore, the lack of clear characteristic symptoms in patients with early-stage lung cancer presents a formidable therapeutic challenge. To date, radiotherapy and chemotherapy have been the two major treatment methods (2,3). However, lung cancer may become resistant to radiotherapy, causing the radiation treatment to be ineffective. Therefore, the exploration of new therapies to enhance the radiosensitivity of lung carcinoma may be of vital clinical significance (4). Ionizing radiation (IR) has been demonstrated to evoke a series of biochemical events inside the cell, including cell cycle arrest, DNA damage and repair, signal transduction and apoptosis (5). However, the mechanism by which cancer cells escape from IR-induced events remains to be elucidated.

The modification of proteins with small ubiquitin-related modifier (SUMO) modulates the substrate’s activation, function and subcellular localization (6). SUMOylation is catalyzed by SUMO-specific activating (E1), conjugating (E2) and ligating (E3) enzymes. SUMOylation is a dynamic process that is reversed by a family of SUMO-specific proteases (SENPs) (7). These enzymes are critical in maintaining a balance between the level of unmodified and SUMOylated proteins that mediate SUMOylation-dependent cellular function (8). In mammalian cells, to date, six SENPs have been identified (SENP1, -2, -3, -5, -6 and -7), which have different substrate specificities and subcellular localizations (9). Among the SENP family, most is understood about SENP1, which is important in placental development and erythropoiesis (10,11). Furthermore, SENP1 has also been revealed to be involved in the development and progression of several types of cancer (12,13). However, while SENP1-specific inhibitors have been designed (14,15), whether SENP1 is a potential drug target for cancer treatment remains unclear.

The present study was conducted to identify markers of radioresistance that may serve as future targets for modulation to enhance the efficacy of radiotherapy. The results of the study showed that the inhibition of SENP1 markedly enhanced the radiosensitivity of lung carcinoma by promoting IR-induced cell cycle arrest, γ-H2AX expression and apoptosis. Thus, these data suggest that SENP1 may be a promising target for enhancing the efficacy of lung carcinoma radiotherapy.

## Materials and methods

### Tissue samples

Primary lung carcinoma and adjacent non-tumor lung tissues were collected during routine therapeutic surgery conducted at the Thoracic Department of the Xuanwu Hospital of Capital Medical University (CMU; Beijing, China). All samples were obtained with informed consent from the patient and with approval from the Thoracic Department of the Xuanwu Hospital of CMU. This study was approved by the ethics committee of Xuanwu Hospital of Capital Medical University (Beijing, China).

### Cell culture

The human lung carcinoma cell line A549 was cultured in Dulbecco’s modified Eagle’s medium (DMEM; Invitrogen Inc., Carlsbad, CA, USA) supplemented with glutamine, penicillin, streptomycin and 10% fetal bovine serum (FBS; Invitrogen Inc.). H460 cells were cultured in RPMI media (Invitrogen Inc.) with 10% FBS. The cells were maintained in a humidified incubator with 5% CO_2_.

### Quantitative polymerase chain reaction (PCR)

RNA was extracted using the mirVana™ miRNA Isolation kit (Applied Biosystems, Invitrogen Life Technologies, Carlsbad, CA, USA). cDNA was synthesized from total RNA using the Taqman miRNA High-Capacity cDNA Reverse Transcription kit (Applied Biosystems) with primers specific to SENP1 or 18S, an endogenous control. Quantitative PCR was performed using the Taqman microRNA PCR system (Applied Biosystems) according to the manufacturer’s instructions. Briefly, cDNA was combined with Taqman Universal PCR Master mix and probes specific for SENP1 or 18S (Applied Biosystems). PCR was performed in 96-well optical plates. SENP1 Ct values were normalized to 18S Ct values and the relative expression was calculated using the −Δ ΔCt method. Primers for SENP1 (forward: TTGGCCAGAGTGCAAATGG; reverse: TCGGCTGTTTCTTGATTTTTGTAA) and the housekeeping 18S rRNA (Applied Biosystems) were used.

### Plasmids and transfection

Human SENP1 was amplified from 293T cDNA library and subcloned into pcDNA-3.1-Myc plasmid. The transient transfections were performed by Lipofectamine 2000 (Invitrogen, Shanghai, China), according to the manufacturer’s instructions.

### RNA interference

Two 21-nucleotide SENP1 small interfering RNAs (siRNAs; si-1: AACTACATCTTCGTGTACCTC and si-2: CTAAACCATCTGAATTGGCTC) and nonspecific siRNA were synthesized (Dharmacon, Thermo Fisher Scientific, Inc., Waltham, MA, USA). The SENP1 and nonspecific siRNA oligos were then inserted into a pSuppressorNeo vector (Imgenex Corp., San Diego, CA, USA), according to the manufacturer’s instructions. Following this, the A549 and H460 cells were transfected with the siRNA plasmid using Lipofectamine 2000 (Invitrogen Life Technologies).

### Flow cytometry

Apoptotic cells were assessed in a flow cytometer (Becton Dickinson, BD Biosciences, Franklin Lakes, NJ, USA) using a fluorescein isothiocyanate (FITC)-labeled Annexin-V and propidium iodide (PI) kit (BD Pharmingen, BD Biosciences).

### Western blot analysis

Cells were lysed in sample solution. Following this, the proteins were separated on 10% sodium dodecyl sulfate-polyacrylamide gel electrophoresis (SDS-PAGE) gels, transferred to nitrocellulose membranes. The membranes were incubated with the primary antibodies at 4°C overnight, prior to being incubated with horseradish peroxidase-conjugated secondary antibodies (Cell signaling Technology, Inc., Danvers, MA, USA) for 1 h at room temperature for detection using the SuperSignal™ West Pico Chemiluminescent Substrate kit (Pierce, Rockford, IL, USA). Anti-β-actin and anti-SENP1 antibodies were purchased from Santa Cruz Biotechnology, Inc. (Santa Cruz, CA, USA), while anti-cleavage caspase 3 and anti-γ-H2AX antibodies were obtained from Cell Signaling Technology, Inc.

### Bromodeoxyuridine (BrdU) assays

Cell proliferation was evaluated using an enzyme-linked immunosorbent assay to determine the incorporation of BrdU during DNA synthesis, in accordance with the manufacturer’s instructions (BrdU Cell Proliferation Assay kit; Beyotime Institute of Biotechnology, Shanghai, China). Briefly, 2×10^3^ cells were plated per well onto 96-well plates. Following 36 h of transfection, cell proliferation was measured. The absorbance of the plates at 450 nm was read using a spectrometer.

### Colony-forming assay

A colony-forming assay was performed to determine the radiosensitivity of cells. A549 cells were digested with 0.25% trypsin, pelleted and then resuspended in 1 ml fresh media. The trypan blue dye exclusion method was used to determine the cell viability. Cells were planted at a density of 4×10^5^ cells/ml in six-well dishes and allowed to attach overnight. Following this, cells were transfected with SENP1 or nonspecific siRNAs for 24 h and then irradiated (0–10 Gy). Ten days subsequent to irradiation, cells were fixed using methyl alcohol and stained with Giemsa staining buffer. A population of >50 cells was counted as one colony, and the number of colonies was expressed as a percentage of the number of mock-irradiated cells. The survival curves were plotted using linear regression analyses. The number of clones was examined using macroscopic observation and the colony forming efficiency (CFE) was calculated with the following formula: CFE = clone number/number of seeded cells.

### Radiation exposure

The cells were seeded into 96-well plates and were treated with a range of radiation doses (0–10 Gy) using a 250 kV orthovoltage unit the following day (Philips, Amsterdam, The Netherlands).

### Statistical analysis

All the results were derived from at least three independent experiments and are presented as the mean ± standard error of the mean. The Student’s t-test was used to compare the differences between two groups. P<0.05 was considered to indicate a statistically significant difference.

## Results

### SENP1 is overexpressed in lung cancer tissues

**Figure 1.**
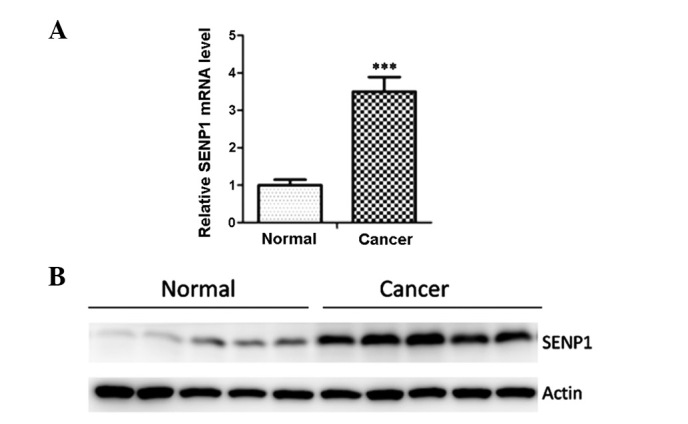
Small ubiquitin-related modifier-specific protease 1 (SENP1) is overexpressed in lung cancer tissues. (A) The mRNA level of SENP1 in 10 paired lung cancer and adjacent non-tumor lung tissues was analyzed using quantitative polymerase chain reaction (PCR). ^***^P<0.001 compared with normal tissues. (B) The protein level of SENP1 in five paired lung cancer and adjacent non-tumor lung tissues was analyzed using western blotting.

SENP1 has been shown to be overexpressed in several types of cancer. To determine whether SENP1 was overexpressed in lung cancer, the mRNA levels of SENP1 in 10 paired lung cancer and adjacent non-tumor lung tissues were assessed. As depicted in Fig. 1A, the mRNA levels of SENP1 were significantly increased in the cancer tissues when compared with the levels in adjacent normal tissues. Western blot analysis with an anti-SENP1 antibody was then employed to examine the SENP1 protein expression in clinical samples, including five primary lung cancer samples and their adjacent normal tissues. The results showed that SENP1 was overexpressed in the lung cancer samples (Fig. 1B).

### SENP1 regulates lung cancer cell proliferation

**Figure 2.**
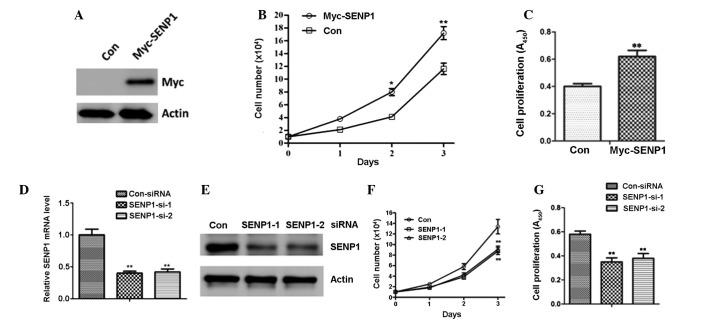
Small ubiquitin-related modifier-specific protease 1 (SENP1) regulates lung cancer cell proliferation. (A) SENP1 expression was determined in H460 cells transfected with empty vector (Con) or SENP1 using western blotting. (B) Growth curve of H460 cells transfected with empty vector (Con) or SENP1. ^*^P<0.05, ^**^P<0.01 compared with Con. (C) The cell proliferative potential [bromodeoxyuridine (BrdU) analysis] was determined in H460 cells transfected with empty vector (Con) or SENP1. The absorption at 450 nm was assayed after transfection for 36 h. ^**^P<0.01 compared with Con. (D) The mRNA level of SENP1 in A549 cells transfected with small interfering RNA (siRNA) oligos targeting SENP1 or scrambled siRNA (Con) was determined using quantitative polymerase chain reaction (PCR). ^**^P<0.01 compared with Con. (E) The protein level of SENP1 in A549 cells transfected with siRNA oligos targeting SENP1 or scrambled siRNA (Con) was determined using western blotting. (F) Growth curve of A549 cells transfected with siRNA oligos targeting SENP1 or scrambled siRNA (Con). ^**^P<0.01 compared with Con. (G) The cell proliferative potential (BrdU) was determined in A549 cells transfected with siRNA oligos targeting SENP1 or scrambled siRNA (Con). The absorption at 450 nm was assayed subsequent to transfection for 36 h. ^**^P<0.01 compared with Con.

To further determine the potential effects of SENP1 in lung cancer cells, H460 cells were transfected with empty vector (Con) or Myc-SENP1 (Fig. 2A). As shown in Fig. 2B, SENP1 overexpression led to a significant increase in the cell number of the H460 cells (Fig. 2B). Moreover, BrdU analysis also suggested that forced SENP1 expression promoted cell proliferation (Fig. 2C). Following this, A549 cells were transfected with siRNA targeting SENP1 to knockdown endogenous SENP1 expression (Fig. 2D and E). As a result of the downregulation of SENP1, there was a marked reduction in the cell number and proliferation of the A549 cells (Fig. 2F and G).

### SENP1-silencing sensitizes lung cancer cells to radiation

**Figure 3.**
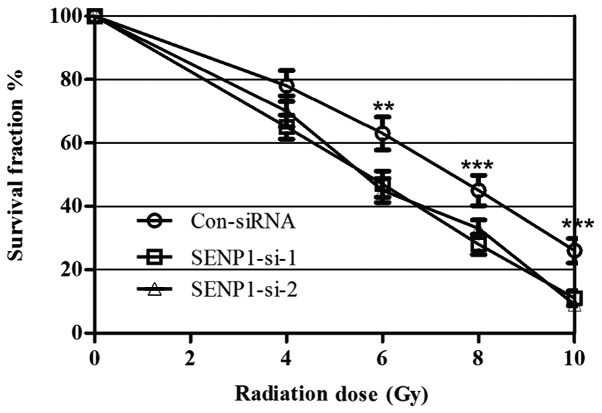
Small ubiquitin-related modifier-specific protease 1 (SENP1)-silencing sensitizes lung cancer cells to radiation. A549 cells transfected with small interfering RNA (siRNA) oligos targeting SENP1 or scrambled siRNA (Con) were treated with 0, 2, 4, 6, 8 and 10 Gy irradiation and survival curves were determined using a colony-forming assay. A Student’s t-test was used to compare the difference in the cell survival fractions between the two groups. ^**^P<0.01, ^***^P<0.001 compared with Con.

To determine whether SENP1 was required for lung cancer cell radioresistance, A549 cells were treated with different doses of IR, from 0 to 10 Gy, following transfection with SENP1 siRNA or nonspecific siRNA. Cell proliferation was subsequently assessed using a colony-forming assay. IR treatment exhibited a dose-dependent inhibitory effect on the growth of the A549 cells, and the SENP1 depletion enhanced this effect, suggesting that inhibition of SENP1 increased A549 cell radio-sensitivity (Fig. 3). Furthermore, identical experiments were performed using H460 cells, which provided similar results to those in the A549 cell line, indicating that SENP1 depletion may increase the radiosensitivity of lung cancer cells.

### Inhibition of SENP1 enhances IR-induced cell cycle arrest, γ-H2AX expression and apoptosis

**Figure 4.**
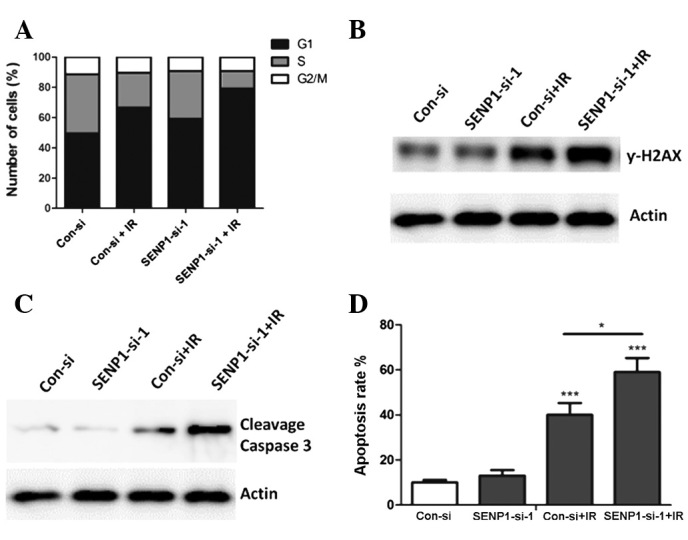
Inhibition of small ubiquitin-related modifier-specific protease 1 (SENP1) enhances ionizing radiation (IR)-induced cell cycle arrest, γ-H2AX expression and apoptosis. A549 cells transfected with small interfering RNA (siRNA) oligos targeting SENP1 (SENP1-si-1) or scrambled siRNA (Con) were treated with or without 10 Gy irradiation. (A) Cell cycle stage, determined using flow cytometry; (B) γ-H2AX expression, determined using western blotting; (C) caspase 3 activation, determined using western blotting with cleavage caspase 3 antibody; (D) apoptosis rate, determined using flow cytometry. ^**^P<0.01, ^***^P<0.001 compared with control siRNA oligos (Con). ^*^P<0.05 compared with control siRNA + IR (Con-si+IR).

The underlying mechanisms that may have caused the SENP1 depletion to increase the radiosensitivity of lung cancer cells were investigated. Since cells in the G1 and G2 stage are sensitive to IR (16), cell cycle analysis was performed to detect whether the cell cycle changed when SENP1-depleted cells were treated with IR. Unlike the nonspecific-siRNA-transfected cells, the SENP1-siRNA-transfected cells treated with IR showed an increased percentage of cells in the G1 stage, suggesting that the enhanced radiosensitivity of the A549 cells may have been due to changes in the cell cycle induced by SENP1 inhibition (Fig. 4A). γ-H2AX has been demonstrated to be positively associated with tumor radiosensitivity (17). Therefore, western blotting was performed to analyze the γ-H2AX expression in the A549 cells following different treatments. SENP1-siRNA-transfected cells treated with IR showed the highest protein level of γ-H2AX (Fig. 4B), suggesting that increased γ-H2AX expression may be one of the underlying mechanisms responsible for the increase in the radiosensitivity of lung cancer cells when SENP1 is inhibited. Furthermore, the ability of SENP1 inhibition to increase IR-induced lung cancer cell apoptosis was investigated. SENP1-siRNA-transfected cells treated with IR were observed to have an increased apoptotic population (Fig. 4D) and an increased activation of caspase 3 (Fig. 4C), suggesting that the increased radiosensitivity may have resulted from an increased rate of apoptosis.

## Discussion

In the present study, it was demonstrated that SENP1 may be a regulator of lung cancer radioresistance. The results showed that SENP1 mRNA and protein was significantly overexpressed in lung cancer tissues when compared with their adjacent non-tumor lung tissues. Moreover, the increased expression of SENP1 in A549 cells significantly increased cell proliferation compared with that of the control A549 cells, and silencing the SENP1 expression in the H460 cell line inhibited cell proliferation. Thus, these data suggested that SENP1 was involved in the regulation of lung cancer cell proliferation.

Following this, the ability of SENP1 inhibition to affect lung cancer radioresistance was examined. It was shown that SENP1 depletion significantly sensitized lung cancer cells to IR and that SENP1 depletion enhanced IR-induced lung cancer cell cycle arrest at the G0/G1 stage. Consistent with this observation, a previous study of SENP1 in colon cancer showed that silencing the expression of SENP1 upregulated the expression of several CDK inhibitors, such as p16, p19, p21 and p27 (18). In addition to these cell cycle regulators, silencing the expression of SENP1 also significantly increased IR-induced γ-H2AX expression, which has been revealed to be positively associated with tumor radiosensitivity. Notably, SENP1 inhibition did not result in lung cancer cell apoptosis, as demonstrated by fluorescence-activated cell sorting (FACS) assay and the caspase 3 activation assay. However, silencing the expression of SENP1 enhanced IR-induced lung cancer cell apoptosis and caspase 3 activation, which may have contributed to the IR-induced lung cancer cell sensitivity.

SENP1 is a deSUMOylation enzyme that has been demonstrated to target a number of key proteins for deSUMOylation (10,19–22). SENP1 has also been revealed to be involved in the development and progression of several types of cancer. In the present study, it was identified that SENP1 was also overexpressed in lung cancer. Recently, specific inhibitors of SENP1 have been designed, and there is consequently a requirement to investigate whether these inhibitors enhance the radiosensitivity of lung carcinoma. It may be of interest to assess whether the deSUMOylation activity of SENP1 is required for the radiosensitization induced by SENP1 knockdown. Further studies are necessary to identify the SUMOylation target of SENP1 that contributes to the radiosensitization induced by SENP1 knockdown.

## References

[1] Sculier JP (2013). Nonsmall cell lung cancer. Eur Respir Rev.

[2] Saadeddin A (2012). Radiotherapy for NSCLC: review of conventional and new treatment techniques. J Infect Public Health.

[3] Hennon MW, Yendamuri S (2012). Advances in lung cancer surgery. J Carcinog.

[4] Nadkar A, Pungaliya C, Drake K (2006). Therapeutic resistance in lung cancer. Expert Opin Drug Metab Toxicol.

[5] Ryan JL (2012). Ionizing radiation: the good, the bad, and the ugly. J Invest Dermatol.

[6] Verger A, Perdomo J, Crossley M (2003). Modification with SUMO. A role in transcriptional regulation. EMBO Rep.

[7] Wang Y, Dasso M (2009). SUMOylation and deSUMOylation at a glance. J Cell Sci.

[8] Yeh ET (2009). SUMOylation and De-SUMOylation: wrestling with life’s processes. J Biol Chem.

[9] Bawa-Khalfe T, Yeh ET (2010). SUMO losing balance: SUMO proteases disrupt SUMO homeostasis to facilitate cancer development and progression. Genes Cancer.

[10] Cheng J, Kang X, Zhang S, Yeh ET (2007). SUMO-specific protease 1 is essential for stabilization of HIF1α during hypoxia. Cell.

[11] Yu L, Ji W, Zhang H (2010). SENP1-mediated GATA1 deSUMOylation is critical for definitive erythropoiesis. J Exp Med.

[12] Bawa-Khalfe T, Cheng J, Lin SH, Ittmann MM, Yeh ET (2010). SENP1 induces prostatic intraepithelial neoplasia through multiple mechanisms. J Biol Chem.

[13] Bettermann K, Benesch M, Weis S, Haybaeck J (2012). SUMOylation in carcinogenesis. Cancer Lett.

[14] Qiao Z, Wang W, Wang L (2011). Design, synthesis, and biological evaluation of benzodiazepine-based SUMO-specific protease 1 inhibitors. Bioorg Med Chem Lett.

[15] Chen Y, Wen D, Huang Z (2012). 2-(4-Chlorophenyl)-2-oxoethyl 4-benzamidobenzoate derivatives, a novel class of SENP1 inhibitors: Virtual screening, synthesis and biological evaluation. Bioorg Med Chem Lett.

[16] Hanai K, Babazono T, Nyumura I (2010). Involvement of visceral fat in the pathogenesis of albuminuria in patients with type 2 diabetes with early stage of nephropathy. Clin Exp Nephrol.

[17] Taneja N, Davis M, Choy JS (2004). Histone H2AX phosphorylation as a predictor of radiosensitivity and target for radiotherapy. J Biol Chem.

[18] Xu Y, Li J, Zuo Y (2011). SUMO-specific protease 1 regulates the in vitro and in vivo growth of colon cancer cells with the upregulated expression of CDK inhibitors. Cancer Lett.

[19] Li X, Lee YK, Jeng JC (2007). Role for KAP1 serine 824 phosphorylation and sumoylation/desumoylation switch in regulating KAP1-mediated transcriptional repression. J Biol Chem.

[20] Yang Y, Fu W, Chen J (2007). SIRT1 sumoylation regulates its deacetylase activity and cellular response to genotoxic stress. Nat Cell Biol.

[21] Lindberg MJ, Popko-Scibor AE, Hansson ML, Wallberg AE (2010). SUMO modification regulates the transcriptional activity of MAML1. FASEB J.

[22] Van Nguyen T, Angkasekwinai P, Dou H (2012). SUMO-specific protease 1 is critical for early lymphoid development through regulation of STAT5 activation. Mol Cell.

